# Towards a Systematic Screening Tool for Quality Assurance and Semiautomatic Fraud Detection for Images in the Life Sciences

**DOI:** 10.1007/s11948-016-9841-7

**Published:** 2016-11-15

**Authors:** Lars Koppers, Holger Wormer, Katja Ickstadt

**Affiliations:** 1 Department of Statistics, TU Dortmund University, Vogelpothsweg 87, 44227 Dortmund, Germany; 2 Institute for Journalism, TU Dortmund University, Emil-Figge-Straße 50, 44227 Dortmund, Germany

**Keywords:** Digital image, Ethics, Manipulation, Image processing, Fraud detection

## Abstract

The quality and authenticity of images is essential for data presentation, especially in the life sciences. Questionable images may often be a first indicator for questionable results, too. Therefore, a tool that uses mathematical methods to detect suspicious images in large image archives can be a helpful instrument to improve quality assurance in publications. As a first step towards a systematic screening tool, especially for journal editors and other staff members who are responsible for quality assurance, such as laboratory supervisors, we propose a basic classification of image manipulation. Based on this classification, we developed and explored some simple algorithms to detect copied areas in images. Using an artificial image and two examples of previously published modified images, we apply quantitative methods such as pixel-wise comparison, a nearest neighbor and a variance algorithm to detect copied-and-pasted areas or duplicated images. We show that our algorithms are able to detect some simple types of image alteration, such as copying and pasting background areas. The variance algorithm detects not only identical, but also very similar areas that differ only by brightness. Further types could, in principle, be implemented in a standardized scanning routine. We detected the copied areas in a proven case of image manipulation in Germany and showed the similarity of two images in a retracted paper from the Kato labs, which has been widely discussed on sites such as pubpeer and retraction watch.

## Introduction

Pictures and images play a key role in the documentation and presentation of results in the life sciences. In cases of fraud, images have often been the key to identifying manipulation and falsification in a scientific work. As a survey by the US Office of Research Integrity (ORI) already showed more than ten years ago, not only the incidence of allegations involving questionable images has increased, but also their incidence relative to other ORI cases (Krueger 2002). Images were also a central issue in cases that garnered broader media attention, such as the Hwang clone fraud case in Korea, the biggest cancer research fraud case by Herrmann/Mertelsmann/Brach in Germany, or the case of former oral cancer research star Jon Sudbø in Norway. In the Hwang case, which is considered “one of the highest profile events in South Korea’s history” (Logan et al. 2010: 172), results such as DNA fingerprinting analyses and photographs of cells in a Science article from 2004 were fabricated (Kakuk 2009: 548). In the German case, 94 publications were found to contain falsified or suspicious data, including many cases of recycling the same images in different contexts and publications, or copying and pasting within a certain image (Couzin and Unger 2006: 39; Abbott and Schwarz 2002). In one of the fraudulent publications from Norway in the prestigious New England Journal of Medicine, one of the paper’s images of mouth lesions was found to be a magnified version of another image in the same article (Couzin and Schirber 2006; for an overview of fraud in oncology: Schraub and Ayed 2010).

Leaving aside such individual and often spectacular cases that have been uncovered, the total number of image manipulations in submitted scientific papers remains unknown and can only be estimated, e.g. by online surveys among scientists. According to such a survey by Martinson et al., 0.3% of 3247 scientists admitted to having “cooked” or falsified research data themselves. About 15% said that they had previously engaged in behaviors such as “dropping observations or data points”, and 4.7% admitted to reusing data in two or more publications (Martinson et al. 2005) (the survey did not explicitly ask about image manipulation). Recently, based on a visual (“by eye”) screening of 20,621 papers in 40 scientific journals, a group of US-researchers estimated the prevalence of the specific case of inappropriate image duplication at 3.8%, with an increasing tendency during the past decade (Bik et al. 2016). This is in line with the observation that in biomedical literature, the number of retractions has increased in the last few years, in many cases due to manipulated images (Krueger 2012). As efficient and systematic screening of image manipulation is not yet available, it can only be assumed that the technical (software) possibilities of image editing may have increased the probability of image manipulation.

Spectacular cases of fraud with broader media attention refuel the debate on how such manipulation could have been avoided and who will be responsible for better quality control in the future. Journal editors have recognized this problem and organized in the “Committee on publication ethics” (COPE, http://publicationethics.org). However, journals such as the Journal of Cell Biology controlling systematically images are rather regarded as an exception (Couzin 2006b). Responsible editors usually point out reviewers’ and editors’ limited possibilities, as did Donald Kennedy, former editor-in-chief at Science: “Peer review cannot detect [fraud] if it is artfully done. (…) And the reported falsifications in the Hwang paper—image manipulation and fake DNA data—are not the sort that reviewers can easily spot” (Couzin 2006a). Concerning the above-mentioned case of oncologist Jon Sudbø, Richard Horton, editor of The Lancet, claimed: “This is all so similar to the Hwang thing that we have just been through. (…) Peer review is a great system for detecting badly done research, but if you have an investigator determined to fabricate an entire study, it is not possible to pick it up” (Butler 2006). Even clearly fabricated papers have a good chance to be accepted, as John Bohannon showed in an experiment with free access journals (Bohannon 2013).

These statements seem to still be true today, at least for more sophisticated manipulations that are undetectable by the bare eye, or for manipulations obviously violating established guidelines such as Nature Journal’s guidelines for “Image integrity and standards” (Nature 2016). Such guidelines provide some orientation to which degree an image may still be regarded as authentic after electronic corrections to brightness, contrast etc. To our knowledge, there is still a lack of widely spread and standardized screening methods for reviewers or editors to routinely verify the authenticity of a submitted scientific image. In principle, such screening tools would be useful for everybody involved in the process of quality control. However, journal editors, in particular, should have a choice from a variety of different methods because falsifiers, who also have access to any given screening tool on the free market, will eventually learn to mask their manipulations and render them undetectable by this specific screening tool.

At least in the case that an image has already been labeled as suspicious, institutions such as ORI offer some tools (called “forensic droplets”) for the examination of “questionable” scientific images (http://ori.hhs.gov/droplets). These tools yield images, but do not offer a measurable or easily comparable result between images (like rankings or probability of manipulation). However, such tools seem to be rather suitable for data that is already questionable, and may be of some help in the daily routines of editors and reviewers. Some software like Rigour^1^ (http://www.suprocktech.com) offers batch processing of images to detect manipulated areas in images. In this work, we explore and discuss a general procedure and basic statistical algorithms as a first step towards a possible automatic routine control of scientific images in the life sciences, and prospectively, beyond.

## Types of Image Manipulation

From a mathematical point of view, according to which images are nothing but a matrix of pixels with different values, the type of potential image manipulation (blots, electrophoretic gels, etc.) is secondary. More important aspects are image characteristics such as color (homogeneous or heterogeneous values inside the matrix), resolution (size of the matrix), etc., which are used to scan for suspicious images. In our approach, we consider images to be data sets that can be systematically scanned for manipulation. Our main goal is to search for similar areas. Therefore, our methods require images without large monochromatic areas in which everything looks similar. Typically, large monochrome areas in themselves are indicative of manipulation or inappropriate post-processing of images (Cromey 2010). On the other hand, large areas of “noisy background” for which copied areas can be searched are extremely valuable. Outside of the background areas, the signal of the image information is usually much stronger (for example dark points on a light background) than the signal coming from a manipulation, making the latter undetectable. Here, we suggest some basic algorithms to detect image manipulation.

A journal’s integrity standards typically define image alteration and manipulation from the author’s perspective. The journals’ image integrity standards usually don’t offer a general and explicit distinction of fraudulent and non-fraudulent (but still unacceptable) image manipulation. For example, Nature’s standards for image integrity (http://www.nature.com/authors/policies/image.html) advise avoiding tools like Adobe Photoshop’s^®^ cloning and healing tools, which alter single areas of an image in a nonlinear way. Global linear transformations (like changes in brightness and contrast) are allowed to a certain degree if they are necessary and mentioned in the description. Other authors distinguish in their digital imaging guidelines between “usually acceptable” (e.g., simple adjustments to the entire image), “questionable” (e.g., manipulations that are specific to one area of an image and are not performed on other areas) and “very questionable” (e.g., cloning or copying objects into a digital image, from other parts of the same image or from a different image) (Cromey 2010). However, the degree to which such transformation is still acceptable, and whether a description of the image treatment is sufficient can only be decided on a case-by-case basis.^2^

If we want to detect questionable manipulation, we have to look at the issue from a data point of view. From this perspective, we do not focus on disallowed alteration methods, but rather on the effects on the data itself. Based on our observations of fraud cases described in the cited literature, we propose the following simple general classification of data manipulation:

- Type 1: Manipulation by deleting unwanted data information (for example using the Photoshop cloning Tool)
- Type 2: Duplication by reusing images in different papers or contexts
- Type 3: Manipulation by adding information/data points.

**Fig. 1:**
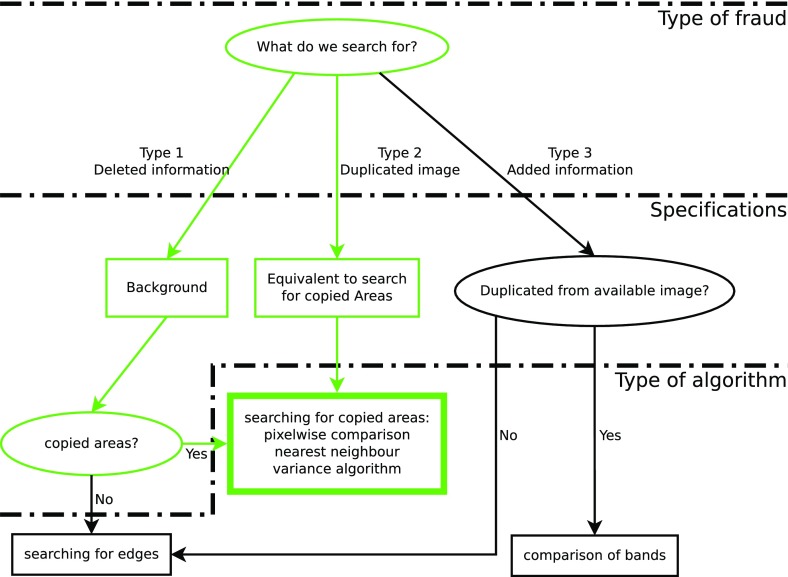
To search for manipulated images, we first decide what type of fraud we anticipate: deleted information, added information or duplicated images (*top row*). In a second step, we specify the manipulation in relation to data availability and use of copied areas (*mid row*). In the last step, we determine the type of algorithm for the actual problem (*bottom row*). The *green pathways* refer to the two cases in which our algorithms contribute to the scanning routine. *Ellipses mark knots* that require a decision, *rectangles* provide additional information

The flow chart in Fig. 1 shows the classification of the three different types of manipulation (on the top) into different detection strategies. The green pathways show the strategies that we examine in our work. At first glance, the second type (duplication in different works) seems to be the most labor-intensive to detect because it requires extensive cross-checking with images that are already published across the entire literature in a given field. In the above-mentioned Herrmann/Mertelsmann/Brach case, investigators often had to rely on their memory; they had seen the questioned image before in another publication by the same author and had to look it up “manually” (Wormer 1999). Today, “post-publication peer review” forums and websites such as “pubpeer” (http://pubpeer.com/) or “Retraction Watch” (http://retractionwatch.com/) seem to be helpful for such examinations, notably after publication. Cases of duplicated images appear on “Retraction Watch” or other platforms on an almost weekly basis. The duplicated images can often be found in the same paper or in other works by the author(s). That reduces the amount of effort required for image comparisons. One recent example is a 2013 paper about human cloning that created some excitement over duplicated images (Tachibana et al. 2012). A few days after publication, an anonymous investigator presented the duplications on “pubpeer”. However, an algorithm to detect duplicated images would have helped the journal detect these images *prior to* publishing.

### Procedures to Detect Images with Added Information

If parts of the information in a given image are added to the original version, this copied-and-pasted area leaves characteristic edges at the border of the copied area. Therefore, it is necessary to spot visible or hidden edges around important image data (e.g. bands in Western Blots) to detect cut points and, in a second step, the origin of the copied information (see Fig. 1, type 3). One problem in detecting suspicious edges is lossy image compression. Most published images use the jpg-format, which employs a lossy compression algorithm based on 8 × 8 pixel blocks (ISO/IEC 10918-1: 1993). When we look at edges, the first step is to discriminate edges caused by compression from edges caused by manipulation. Again, manipulation type 3 is difficult to process automatically because the signal from the added data is typically much stronger than the signal from the edges of copied areas. As our goal is to outline the first steps towards a tool for use by journal editors and/or reviewers as a possible screening method of incoming images, this paper will focus on the first two types of possible manipulations (deletion or duplication of areas). Searching for edges requires other types of algorithms which are not the subject of this work. One way to avoid manipulation type 3 in the future would be for journals to accept only uncompressed image data at submission for quality checks.

### Procedures to Detect Deleted Information and Duplicated Images

At first glance, searching for deleted information in a given image seems to be a paradox: How to look for something which is not there anymore? Typically, the deleted information has been replaced by background noise. This can be done by copying and pasting another part of the image in a way that hides the unwanted area (compare the specification step in the flow chart in Fig. 1). Since we cannot search for the deleted data, we must search for the origin of the copied background. In principle, it is possible to detect deleted information by searching for edges, but the above-mentioned problem of compressed images applies here, too.

One proposed method to detect areas with data deletion is to search for background regions which differ from their direct neighborhood in the image, e.g. by changes in luminance or color. An alternative is to search for similar areas, which are indicative of data manipulation by copying and pasting. In this work, we considered data deletion by replacement with background. In a second step, we examined a related problem: finding identical images or identical details.

One strategy to match a copied region to its new environment is changing contrast. After such a change, the copied area is no longer identical with its original. For that reason, we also need an algorithm to detect regions that are similar, although modified.

## Methods

We provided three different algorithms to detect copied areas in the background. In this section, we first describe data pre-processing, followed by the three algorithms. As a last step, we present a tool to summarize the results. The algorithms are all part of a newly developed R (R Core Team 2015) software package FraudDetTools, which is available from the authors. The package contains a selection of functions written in R. All algorithms work with one or two different images. The package has two core functions: The function readImage collects the pre-processing steps; nN9Var provides the different comparison algorithms. In addition, some functions that output results and some sample data are also part of FraudDetTool.

### Data Pre-processing

Depending on the origin of the data that is to be analyzed, some pre-processing steps are necessary. Images can be easily read as JPEG- or PNG- formats. To isolate parts of an image or an image from a bigger figure, the data must be handled with care to prevent data alteration. Formats like JPEG are lossy in their data compression. To avoid data loss, they have to be saved in lossless formats like PNG. To analyze the images, we transform them into an image matrix. Our package includes the function readImage, which uses the two R packages *jpeg* and *png* (Urbanek 2013a, b) to create those image matrices and additionally transforms color images into grayscale ones. The image matrix is the basis for all following analyses. Every entry represents one pixel of the original image. The matrix values range from 0 to 1. For a typical 8-bit image, there are 256 possible values. For monochrome areas in the picture, a second image matrix has to be created. The matrix values corresponding to the monochrome areas have to be changed to a new, unique value to prevent false positive matches. Typically, white (1) and black (0) are the values which include monochrome areas. Even after this preparation, the variance algorithm does not work for images that include monochrome areas.

### Comparison Algorithms

The two images (two different images, or the original image and a (pre-processed) copy) are compared in any possible shift. The parts of the image that do not overlap are compared with the pixels on the other side of the image: e.g., a shift by one line causes the first line of the first image and the last line of the second image to be compared. If we compare images of different sizes, only the range of the smaller image is used. This procedure is the same for all three algorithms.

For a pixel-wise comparison, we count the number of identical superimposed pixels. The nearest neighbor algorithm counts identical 3 × 3 pixel blocks. The variance algorithm computes the variances in every 3 × 3 pixel block and accumulates them for the whole image. All algorithms create a result matrix which contains the results for every shift. The index of the matrix rows and columns indicates a shift by this number of rows and columns. For 3 × 3 pixel blocks, the entry of the result is at the position of the top left pixel.

### Localization of Similar Areas

The result matrices of the three algorithms show the number of identical/similar pixel/neighborhoods or the sum of neighborhood variances, respectively, for every shift. An additional approach provides localization matrices. These are implemented for the nearest neighbor and the variance algorithm. Every entry counts the number of identical nearest neighbor areas or variances below the cut-point, respectively, over all shifts. Thus, localization matrices help finding areas with a large number of identities in an image, see visualization in Fig. 5c.

## Examples

To test our algorithms, we used three different types of data: A test image for such procedures and two real manipulation cases. The first real data example is a simple copy-and-paste manipulation of type 1, the second a more difficult manipulation of type 2 including some data alteration. Despite the fact that they are manipulated, reproduction of the manipulated images is necessary to show the results of our algorithm.

### Example 1: ORI Test Image

First, we explored the algorithms on a test image from the ORI (http://ori.dhhs.gov/) consisting of weak background noise. This image was designed by the ORI to test new routines to search for copied areas. We employed all three algorithms to the whole image to find the copied regions. As one would expect for 250,000 pixels and 256 shades of grey, there are many identical pixels. For the ORI test image, every shift has at least 15,020 identical pixel pairs (pixel-wise comparison). If we count an *identity* only if the nearest neighborhood of 8 pixels including the origin pixels themselves is identical (nearest neighbor), the number of identities for every shift is between 0 and 2187. We are interested in shifts containing a larger number of identical pixel pairs (or 3 × 3 areas) relative to most of the other shifts to avoid random matches. The absolute number of identities is secondary.

**Fig. 2:**
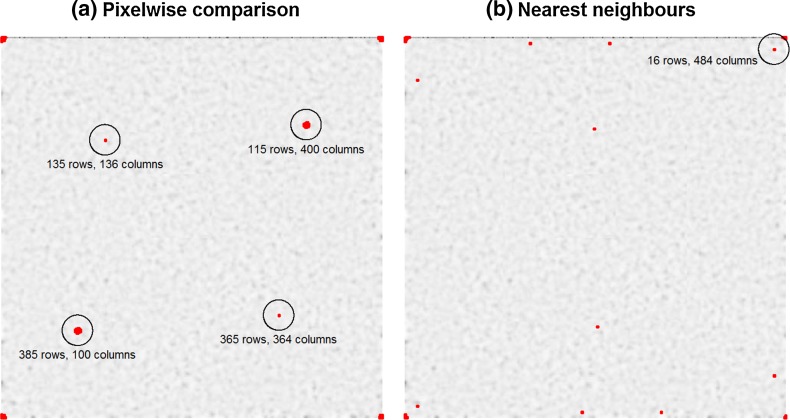
The images show shifts, including the most pair-wise identical pixels. The background consists of the original ORI image (8-bit grayscale image with 500 to 500 pixels). The left image marks shifts including more than 17,000 identical pixels. Every *red mark* represents the top left edge of a shift. *Big marks* represent more than one conspicuous shift in a small area. The right-hand image visualizes shifts including more than 70 identical (3 × 3) pixel areas. The *red marks* in the edges of both images result from very small shifts and the similarity of the neighborhood of a pixel to itself and are not of interest. Figure **a** shows four interesting shifts marked by *black circles*. The coordinates refer to the shift which includes the most identical pixels (shifts in relation to the *top left corner*). In figure **b**, only the shift referring to Fig. 4 is marked

Figure 2 shows the shifts containing the most identical pixels. Most of them are shifted by only a few pixels (see marks in the edges of the image). For these cases, the reason for the many identities is the similarity of the neighbor pixels in the original image. To obtain the really interesting shifts, we had to filter the results. The shifts of interest are those in which the image is shifted more than a few pixels and which contain a large number of identical pixels or 3 × 3 areas. For the test image, there are two conspicuous shifts.

**Fig. 3:**
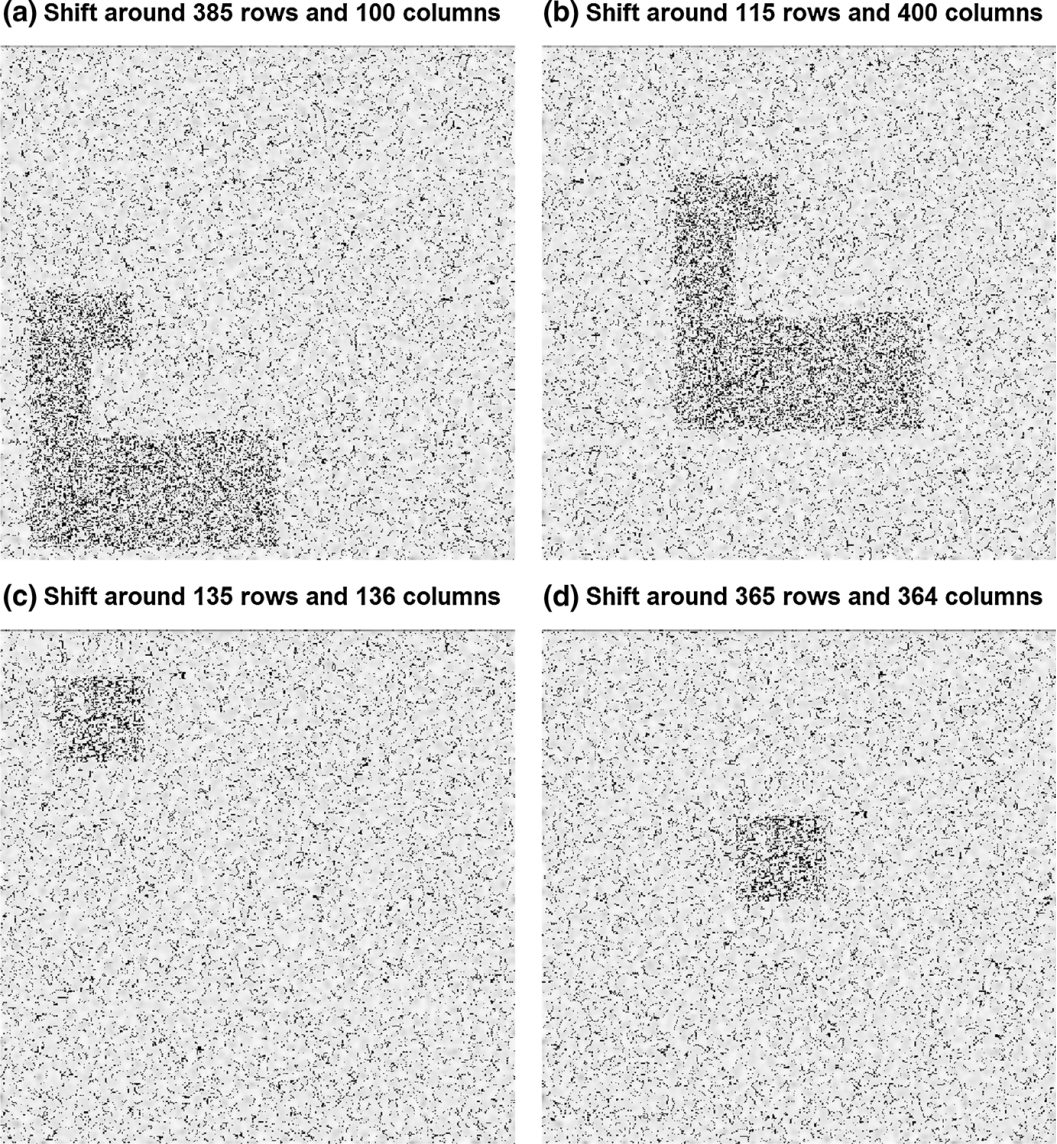
The four conspicuous shifts found by pixel-wise comparison (*black circles* in Fig. 2a). Figures **a** and **b** as well as **c** and **d** belong together (we shift **a** over **b**, or **b** over **a**). The shifts by 385 rows and 100 columns and 115 rows and 400 columns, respectively, have 22,889 identical pixels. The shifts by 135 rows and 136 columns and 365 rows and 364 columns, respectively, have 17,177 identical pixels. In both cases, there is an area containing more identical pixels than outside. The nearest neighbor algorithm cannot detect these areas because of missing identical 3 × 3 areas. Background image: ORI test image

**Fig. 4:**
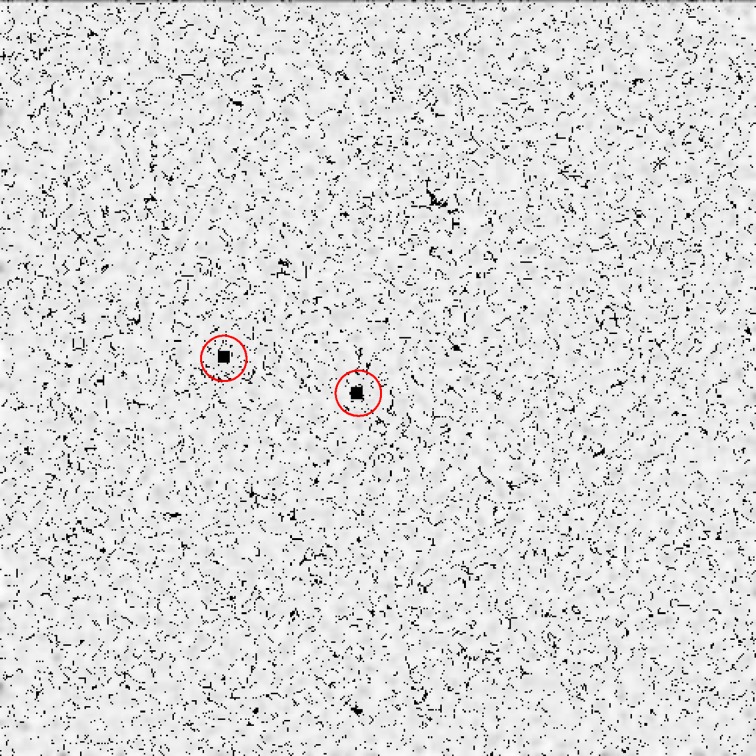
Identical pixels for the shift by 16 rows and 484 columns (*black circle* in Fig. 2b). Most of the identical pixels cannot be detected by the nearest neighbor algorithm, only the two identical areas are visible to this algorithm (see *red circles*). The pixel-wise comparison algorithm does not detect this shift because of the low signal-to-noise ratio. Background image: ORI test image

In Fig. 3, the identical pixels for these shifts are marked. In both cases, there is a region in which the number of identical pixels is much higher than outside the region. Every two shifts belong together, representing the two similar areas. Only the pixel-wise comparison algorithm is able to detect these shifts; for the nearest neighbor algorithm, these similarities are impossible to detect, because there are no identical pixels in which the nearest neighbors (3 × 3 areas) are all identical, too. The third algorithm, the variance algorithm, only detects the bigger area. The signal from the small square cannot be differentiated from random hits. The nearest neighbor algorithm also detects some shifts including small identical areas, as shown in Fig. 4. For these findings it is important to check the original shades of grey in these areas. If they are all the same, due to a monochrome area in the image or a large image compression, identical areas can more easily be found by coincidence than in a high-contrast area or non-compressed image.

Using our algorithms, we obtained different types of similar areas in the ORI test image. This example has shown that our algorithms can work on test data. Next, we looked for applicability in a real life example that had already been identified as a manipulation.

### Example 2: Copied Areas

The nearest neighbor algorithm only finds simple copy-and-paste shifts. Nevertheless, it seems to be suitable for practical image analysis. We demonstrated this using an established case with a simple copy-and-paste manipulation as an example. In 1998, Noé and Breer published the paper “Functional and Molecular Characterization of Individual Olfactory Neurons” (Noé and Breer 1998). Five years later, the German Research Foundation (DFG) ascertained that two figures in this publication were manipulated (DFG 2003). According to this report, the authors had replaced the primer bands of the electrophoresis gels with background. We applied our algorithms to one electrophoresis gel from Fig. 6b in the cited paper. In the original image from the research paper, our algorithms cannot detect the copied areas because of low image quality. The image quality of the corrigendum is much better than the image in the original paper. On the image data that was extracted from the corrigendum, the algorithms work very well.

**Fig. 5:**
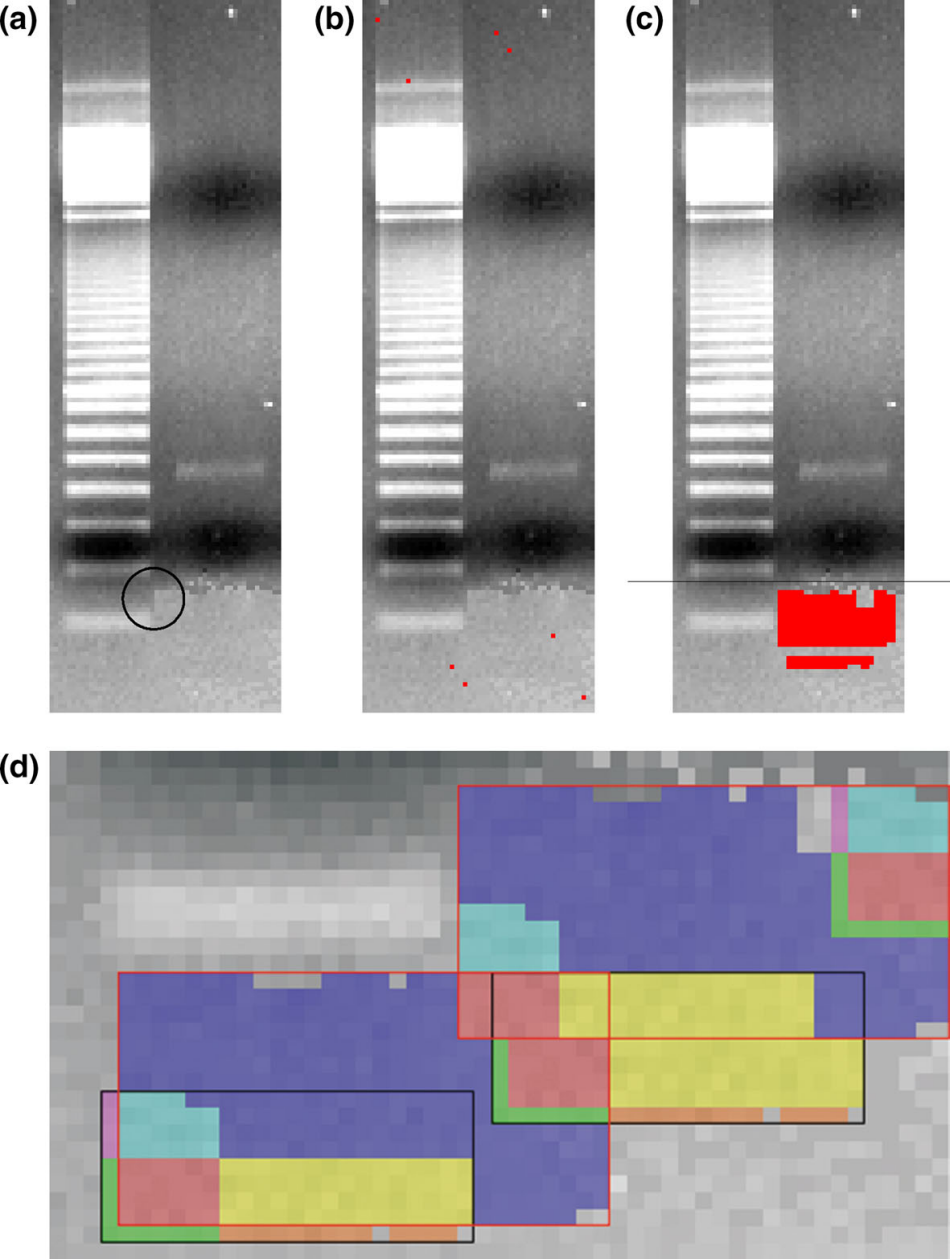
The Western Blot from Fig. 6b from Noé and Breer (1998) (in the version of the corrigendum). The primer bands where replaced by background. A visible edge of the insertion is marked by the *black circle* in figure **a**. The *red dots* in b show the eight shifts including identical (3 × 3) neighborhoods. All these shifts refer to the copied red area in figure **c**. This figure shows the location of the *top left pixel* from an identical (3 × 3) neighborhood for all shifts (*red marks*). Due to the nearest neighbor algorithm, only one of the identical neighborhood pairs is marked (see *red area*). The *black line* separates the last 30 pixel rows, which are shown in detail in figure **d**. Identical areas are marked in the *same color*. The *two rectangles* (black-rimmed and red-rimmed) show the necessary shifts to create these image manipulations. The first shift was the copy-and-paste of the black-rimmed rectangle. *Both rectangles* were copied from left to right. Only eleven pixels in the target area cannot be explained by these shifts (the unmarked ones without the unmarked 4 × 2 pixel-block. Background image: Noé and Breer (1998) corrigendum

The edges of a copied area at the bottom of the right band are visible to the naked eye (panel ‘a’ in Fig. 5), yet the origin of the copied area is not. If we compare this block (29 × 9 pixels) to every other part of the image, we find some identical areas. Additional information about the location of the copied area is not necessary. In a second step, we tried to detect copied areas without any information about their location. To run the algorithms on the whole image, some pre-processing for monochrome areas is necessary because of the light image areas (see “Methods”). The nearest neighbor algorithm exactly points out those shifts, which are necessary for this manipulation (see panel ‘b’ in Fig. 5). There are no false positives. To show the image manipulation, the next step is to visualize the identical areas found by the algorithm as shown in panel ‘d’ in Fig. 5. In this case, it is possible to retrace the steps which were likely taken to manipulate the image. It appears that the first step was to copy and paste the black rectangle from the bottom left to the middle. In a second step, the same procedure was executed for the red rectangle, which was modified by the first manipulation. Only eleven pixels inside the copied areas remain which cannot be explained with these two shifts of rectangles.

This example has shown that our algorithms are able to retrace previously identified manipulations. In this case, they also provide additional information (in comparison to the naked eye) about the origin of the copied areas.

### Example 3: Detecting Duplicated Images

The third example consists of two images from a letter in Nature Cell Biology (Suzawa et al. 2003).

**Fig. 6:**
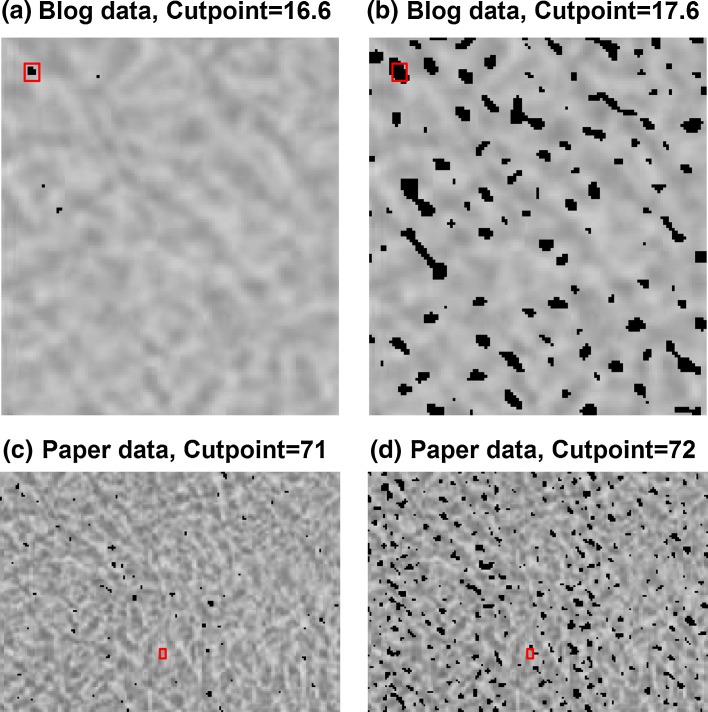
Figures **a**–**d** show results from the variance algorithm. All shifts containing a block variance sum below the cut-point are marked by *black areas*. Figures **a** and **b** show the results from the “11jigen” blog image, figures **c** and **d** are based on the results of the original Kato paper image. The results of the algorithm are compared to the approximate results obtained by visualisation (marked by a *red rectangle*). The variance algorithm detects plausible shifts in the blog data, but not in the original paper data. Background image: Suzawa et al. (2003)

This letter is part of the investigations of the Kato group, on which Retraction Watch has reported frequently (http://retractionwatch.com/?s=Kato). We chose this example because of the rising relevance of blogs reporting on suspicions of scientific misconduct. A screening instrument to verify such allegations would also be useful in this context. In our concrete case, an anonymous whistleblower made allegations in a blog in 2012 (http://katolab-imagefraud.blogspot.de) and also published a YouTube Video (https://www.youtube.com/watch?v=FXaOqwanWnU), pointing out dozens of reused images. Meanwhile, over 40 papers have been retracted. The paper we consider was retracted in November 2014 (Suzawa et al. 2014). We focus on two images of Fig. 2f of the original paper and in the blog. Overlaying the two images manually, we can detect similar structures. For the manual analysis, it does not matter if the data came from the original letter or the blog. For our algorithms, however, the two data sources deliver different results: In the blog data, it is easy to find the correct shift to superimpose the images (see upper two images in Fig. 6). The blog data is a detail of the original image. The images are not exactly identical to the images from the letter. Testing our algorithm on the original data (or on a detail comparable to the blog), we find no corresponding shift (see lower two images in Fig. 6). The cause of the algorithm’s failure is probably a change in scale between the images. The interesting detail in the first image is a few pixels larger than in the second one. The algorithms do not yet feature a scaling correction, so it is impossible to find the correct shifts.

## Discussion

There are many ways to manipulate and reuse images. Developing a screening tool to detect such manipulation requires a systematic classification. Our proposed typology of 3 types of image manipulation may be regarded as a first and useful step for a screening procedure beyond graphical output. With the presented algorithms, we can detect identical areas, large areas which include more identical pixels than expected, and identical areas whose image values are shifted by a constant. However, the detection algorithms cover only a small range of possible manipulations. Our ultimate goal is to create an automated procedure for quality assurance. This will require extending the algorithms and making them sensitive to rotated and scaled images. At this point, the pixel-wise comparison and nearest neighbor algorithms only detect exact identical pixels and 3 × 3 areas, respectively. The nearest neighbor algorithm is more sensitive to small copied areas, whereas the pixel-wise algorithm cannot detect such signals due to the high number of randomly identical pixels. Changes in scale or image quality (e.g. JPEG-compression) render manipulations undetectable to the algorithms. In the original image from the discussed Noé/Breer paper, our algorithms are unable to detect the copied areas because of low quality and changes caused by image compression. The original images from the Kato paper vary minimally in size, also causing the algorithms to fail. However, the tools are a useful addition to the range of existing screening methods and lead to a monitoring system which looks for “outliers” in a collection of images.

Our example shows that it is necessary to use images of good quality. Some journals like the Nature Publishing Group employ the good practice of handling raw data: “In fact, our journals have plans to make this data available to readers, and we expect this measure to increase the overall quality and integrity of the scientific record” (Retraction Blues 2013). This data is important in order to discover manipulated data. Publication of high-quality (raw) data gives scientists the chance to test images using their own procedures, which, of course, is no substitute for a careful image check by the journals.

This is in line with the conclusions derived from our examples. Although more pixels cause longer runtimes for the algorithms, more detail increases the chance of detecting duplicated areas. Lossy image compression should be avoided to ensure correct data representation. The algorithms are too slow to search for duplications in big image archives, but other, more powerful algorithms do exist. However, it is possible to compare all images within a given paper and, and for cases like Sudbø or Herrmann/Mertelsmann/Brach introduced earlier, it is also useful. The algorithms can be part of the quality control routine to avoid duplicating images by mistake. The duplicated images in the recent Tachibana cloning paper cannot be detected at this stage due to incompatibility and changes in scale, but an improved algorithm should be able to manage this type of duplication.

In summary, we can state that all three algorithms are helpful tools for scanning suspicious images. As a next step, they must be supplemented by algorithms which work for rescaled and rotated images. Furthermore, faster implementation is desirable to address the runtime problem. In addition to existing approaches (expert eye and Photoshop procedures), our procedure can generally be used to automatically check large image archives and filter out suspicious images for a precise expert check. To increase the level of automatization, filtering of unusual results (outliers) is possible.

We manually monitored the retractions appearing on “Retraction Watch” for six months, which led us to the assumption that most undetected image manipulation could be avoided if publishers/editors implemented a routine check for the described manipulation. Including the features of our and other algorithms, the next step could be to create a classifier which helps scan for suspicious images. Up to now, the algorithms were tested on examples and on original data from known cases of fraud. For statistical inference, it would be preferable to simulate and model types of image manipulation. The use of algorithms calls for a check of the algorithm itself. Since it is not appropriate to blindly trust a screening tool, we have to investigate the precision and recall of our tools (Rossner 2008).

The goal of this study was to develop a systematic approach to classify different kinds of image manipulation in a suitable form, which can be handled with the basic algorithms we have developed. The proposed classification may also be a means to sharpen awareness of how images should be treated in scientific teaching. As a next step towards using the tool in practice, a quality check by a double blind controlled trial, as recommended by one of our reviewers, is inevitable. However, a set of algorithms that detects suspicious images will have to be continuously extended because image manipulators will continue to find new methods, as well.

Finally, we must point out that an automated scan for suspicious images does not imply an automated judgment. The final decision should always be made by human experts to avoid false positives, but comparison algorithms should support the discussion by providing an initial quality check. Once an algorithm detects a suspicious image, further investigation like the proceeding described in the COPE Flowcharts (publicationethics.org/resources/flowcharts) about fabricated data will be necessary.

## Footnotes

1 Rigour is a closed-source software; public information on the approaches it uses is not available. However, tutorials suggest that this program’s output is processed images.

2 Further questions, such as a more general definition of fraudulent and non-fraudulent, but still unacceptable treatment of images, touch on the broad topic of authenticity, which cannot be discussed in detail in this article. However, many questions in this context have already been brought up in classical works such as Walter Benjamin’s “The Work of Art in the Age of Mechanical Reproduction” and seem to be more important than ever in the digital age.
